# Development of microsatellite markers by transcriptome sequencing in two species of *Amorphophallus* (Araceae)

**DOI:** 10.1186/1471-2164-14-490

**Published:** 2013-07-19

**Authors:** Xingfei Zheng, Cheng Pan, Ying Diao, Yongning You, Chaozhu Yang, Zhongli Hu

**Affiliations:** 1 State Key Laboratory of Hybrid Rice, College of Life Sciences, Wuhan University, Wuhan, 430072 Hubei, PR China; 2 College of Forestry and Life Sciences, Chongqing University of Arts and Sciences, 402160 Yongchuan, Chongqing, PR China; 3 Agricultural Science Academy of Enshi Autonomous Prefecture, Enshi, Hubei, 445002, PR China

**Keywords:** Amorphophallus, Microsatellite marker, Transcriptome, Genetic diversity

## Abstract

**Background:**

*Amorphophallus* is a genus of perennial plants widely distributed in the tropics or subtropics of West Africa and South Asia. Its corms contain a high level of water-soluble glucomannan; therefore, it has long been used as a medicinal herb and food source. Genetic studies of *Amorphophallus* have been hindered by a lack of genetic markers. A large number of molecular markers are required for genetic diversity study and improving disease resistance in *Amorphophallus*. Here, we report large scale of transcriptome sequencing of two species: *Amorphophallus konjac* and *Amorphophallus bulbifer* using deep sequencing technology, and microsatellite (SSR) markers were identified based on these transcriptome sequences.

**Results:**

cDNAs of *A. konjac* and *A. bulbifer* were sequenced using Illumina HiSeq™ 2000 sequencing technology. A total of 135,822 non-redundant unigenes were assembled from about 9.66 gigabases, and 19,596 SSRs were identified in 16,027 non-redundant unigenes. Di-nucleotide SSRs were the most abundant motif (61.6%), followed by tri- (30.3%), tetra- (5.6%), penta- (1.5%), and hexa-nucleotides (1%) repeats. The top di- and tri-nucleotide repeat motifs included AG/CT (45.2%) and AGG/CCT (7.1%), respectively. A total of 10,754 primer pairs were designed for marker development. Of these, 320 primers were synthesized and used for validation of amplification and assessment of polymorphisms in 25 individual plants. The total of 275 primer pairs yielded PCR amplification products, of which 205 were polymorphic. The number of alleles ranged from 2 to 14 and the polymorphism information content valued ranged from 0.10 to 0.90. Genetic diversity analysis was done using 177 highly polymorphic SSR markers. A phenogram based on Jaccard’s similarity coefficients was constructed, which showed a distinct cluster of 25 *Amorphophallus* individuals.

**Conclusion:**

A total of 10,754 SSR markers have been identified in *Amorphophallus* using transcriptome sequencing. One hundred and seventy-seven polymorphic markers were successfully validated in 25 individuals. The large number of genetic markers developed in the present study should contribute greatly to research into genetic diversity and germplasm characterization in *Amorphophallus*.

## Background

*Amorphophallus* is a genus of perennial plants with an underground stem in the form of a corm and an umbrella-shaped leaf blade, which belongs to the family of Araceae, order of Alismatales [1,2]. The genus includes more than 170 species, which are mainly distributed in the tropics or subtropics of West Africa and South Asia [3]. *Amorphophallus* has been cultivated and utilized in China as a medicinal herb and food source for 2000 years. *Amorphophallus* contains abundant water-soluble dietary fiber known as glucomannan, which is beneficial to the human health, possibly being responsible for lowering lipids, systolic blood pressure and glycemia [4,5]. Thus *Amorphophallus* has great potential for commercial application. The diploid species *A. konjac*[6] is widely planted in China, its corm contained high-level glucomannan and possessed wide adaptability, but poor disease resistance and low coefficient of propagation [7]; in the contrast, *A. bulbifer* is a triploid (2n = 39) [6] with high disease resistance, characteristics of multi-seeding relay growth and high coefficient of propagation [8]. However, molecular genetic research of *Amorphophallus* is limited. Many studies have focused on extracting bioactive components [9], and some studies have attempted to identify *Amorphophallus* species taxonomically and to assess the genetic diversity of *Amorphophallus*[10-14]. However, few genomic molecular makers have been developed until now: only 32 microsatellite (SSR) markers were exploited by Santosa *et al.* and Pan *et al.*[15,16]. SSR markers have been shown to be an effective tool to carry out germplasm characterization and genetic diversity studies [17-21]. Thus, more markers are needed for an in-depth understanding of the natural diversity of *Amorphophallus* and to develop strategies for their sustainable utilization. Finally, as far as we know, gene discovery and molecular breeding of new *Amorphophallus* economic cultivars have not been initiated because of the lack of genetic and genomic information.

SSR makers have been widely used in population genetics and crop breeding [22]. According to the original sequences used for development of SSRs, SSRs were divided in two categories: genomic-SSRs, identified from random genomic sequences, and expressed sequence tag (EST)-SSRs, identified from transcribed RNA sequences. Compared with EST-SSRs, the development of genomic-SSRs is very expensive, labor intensive and timing consuming. Genomic-SSRs have uncertain linkage to the transcribed regions of the genome, while EST-SSRs are potentially linked with particular transcriptional regions that contribute to agronomic phenotypes [23,24]. In addition, EST-SSRs have high transferability, because sequences containing EST-SSRs are more conserved than sequences containing genomic-SSRs and can be utilized in other related species [25]. Large numbers of EST sequences produced by deep sequencing technology have been deposited in the NCBI database and are being used for DNA fingerprinting, comparative mapping, and evolutionary analysis [26,27]. However, only a limited number of *Amorphophallus* EST sequence are available in the NCBI database.

Here, we report the generation of a large expressed sequence dataset based on Illumina HiSeq™ 2000 sequencing data from the young leaves of two *Amorphophallus* species, *A. konjac* and *A. bulbifer*. In addition, we developed EST-SSR markers and described their transferability in 14 species and 5 wild unknown “species”. The relationship among cultivars and wild individuals was investigated using the developed EST-SSRs.

## Results

### Assembly of *Amorphophallus* transcriptome data from Illumina sequencing

**Figure 1:**
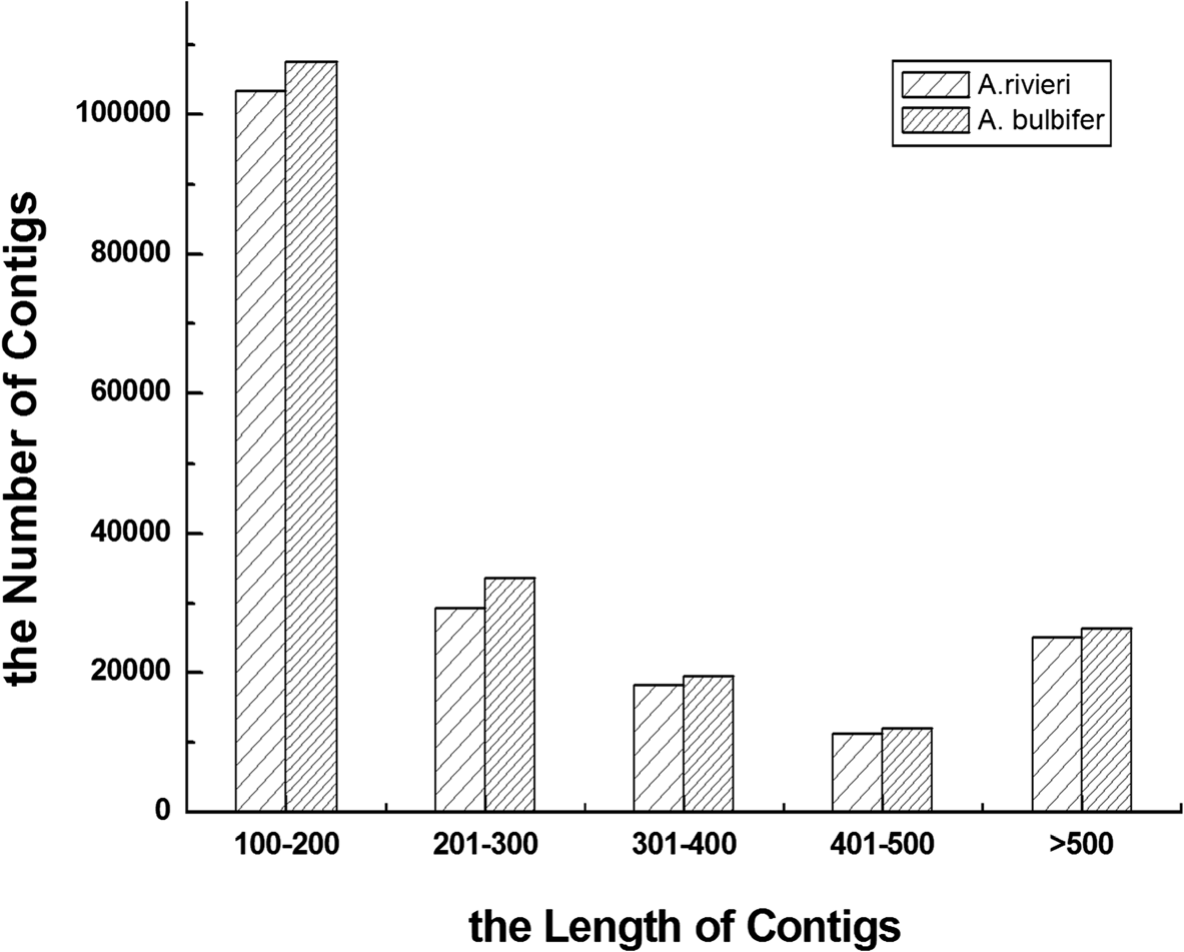
**Frequency distribution of the contig sizes from two *Amorphophallus *species.** The frequency distribution of contig sizes resulting from Illumina HiSeq™ 2000 sequencing, as analyzed using the Trinity software.

After stringent quality assessment and data filtering, Illumina HiSeq™ 2000 sequencing produced 54,986,020 reads for *A. konjac* and 52,334,098 reads for *A. bulbifer* with 92.27% Q20 bases and 93.48% Q20 bases (those with a base quality greater than 20), respectively (Table 1). The total length of the reads was about 9.66 gigabases (Gb). The raw data files are available in the Sequence Read Archive at the National Center for Biotechnology information (NCBI), with accession number SRA057020. Using the Trinity assembler software [28], short-read sequences from *A. konjac* and *A. bulbifer* were assembled into 187,459 contigs and 199,259 contigs, respectively (Table 1). The size distribution of these contigs is shown in Figure 1. Little difference was found in the contig lengths from these two *Amorphophallus* species; the contigs produced from both *A. konjac* and *A. bulbifer* were, on average, 276 bp long, with 381 bp and 372 bp median contig lengths (N50), respectively.

**Table 1:** **Transcriptome reads and assembled contig information for two *Amorphophallus *species**

**Species**	**Total reads**	**Total clean Nucleotides (Nt)**	**Q20 percentage**	**GC percentage**	**Total number of contigs**	**Total length of contigs (Nt)**	**N50 of contigs**	**Mean (Nt)**
*A. konjac*	54,986,020	4,948,741,800	92.27%	56.93%	187,459	51,822,856	381	276
*A. bulbifer*	52,334,098	4,710,068,820	93.48%	54.29%	199,257	54,982,674	372	276

**Figure 2:**
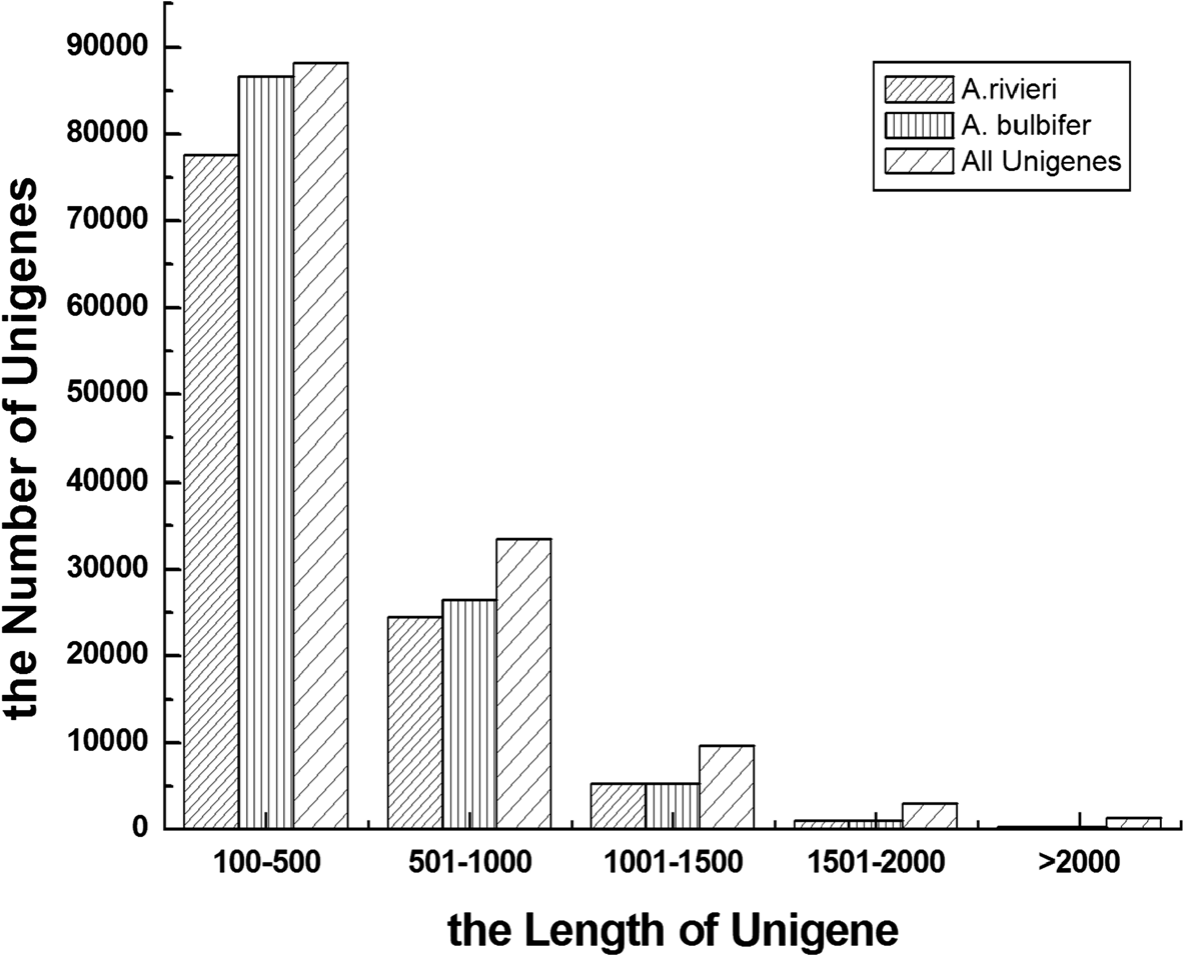
**Size distribution of unigenes from two *Amorphophallus *species.** The frequency distribution of unigene sizes from *A. konjac* and *A. bulbifer* and the combined unigenes, as analyzed with the Trinity software.

With paired-end reads, contigs can be identified from the same transcript and the distance between these contigs can be estimated. Trinity can be used to map the reads back to the contigs, and to connect the contigs into unigenes that cannot be further extended either end. As a result, 108,651 and 119,678 unigenes were obtained, with N50 values of 534 and 524 bp, from *A. konjac* and *A. bulbifer*, respectively (Table 2). To expand the utility of Illumina sequencing data, the contigs from the two species were pooled and assembled into a non-redundant unigene set for further analysis. As shown in Table 2, 135,822 non-redundant unigenes with an average length of 523 bp and an N50 of 635 bp, ranging from 271 to 2,431 bp, were obtained. The length distribution of two species and combined non-redundant unigenes are shown in Figure 2. The results revealed that the contig and unigene size distributions for two species were consistent, which implied the Illumina sequencing solution was reliable and appropriate. Among combined non-redundant unigenes, the length of 88,155 (64.90%) ranged from 100 to 500 bp, 33,529 unigenes (24.69%) ranged from 500 to 1000 bp, and 14,138 unigenes (10.41%) were more than 1000 bp in length.

**Table 2:** **Summary of the unigenes from two *Amorphophallus *species**

**Unigenes source**	**Total number of unigenes**	**Total length of unigenes (Nt)**	**Mean length of unigenes (Nt)**	**N50 of unigenes**
*A. konjac*	108,651	47,721,327	439	534
*A. bulbifer*	119,678	51,480,410	430	524
All	135,822	71,092,160	523	635

### Frequency and distribution of different types of SSR markers

**Figure 3:**
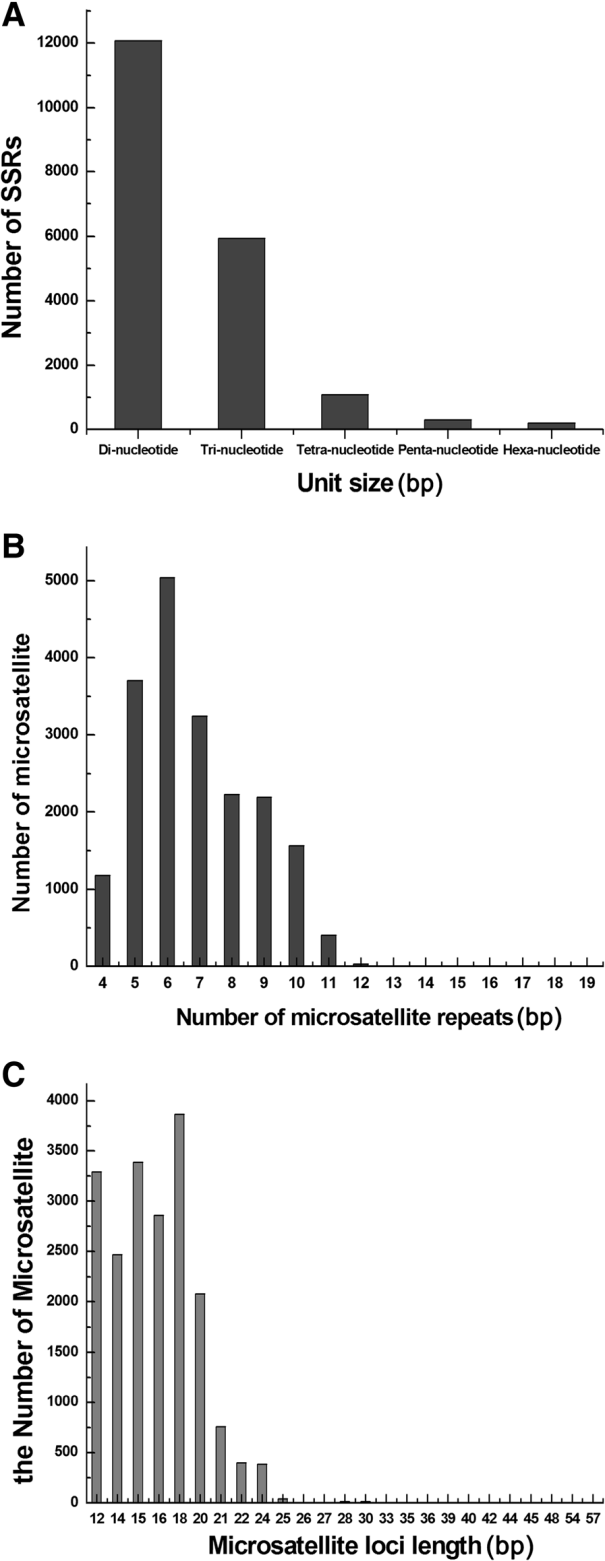
**Frequency distribution of the *Amorphophallus *EST-SSRs of different sizes. A**: Unit size; **B**: Number of repeats; **C**: SSR loci length.

Using a perl script known as MISA, 19,596 SSR loci were detected from 16,027 non-redundant unigenes, representing 11.8% of 135,822 unigenes. A total of 2,840 unigene sequences contained more than one SSR loci. In this study, the mononucleotide repeats were not considered. The probability of SSRs from combined non-redundant unigenes was 14.4% and the distribution density was one SSR loci per 3.63kb. Dinucleotide repeats were the most common type, with a frequency of 61.6% (12,069), followed by trinucleotide (30.3%, 5,943), tetranucleotide (5.6%, 1,083), pentanucleotide (1.5%, 300) and hexanucleotide repeats (1%, 201) (Figure 3A). The combined number of SSR loci from both dinucleotide and trinucleotide repeats was 18,012 with a frequency of 91.9%. The frequencies of SSR distributed in different reiteration numbers are shown in Figure 3B. The number of SSR repeats ranged from 4 to 19, and SSRs with six repeats were the most abundant, followed by those with five tandem repeats, seven tandem repeats and eight random repeats. Motifs that showed more than 12 reiterations were very rare, with a frequency of <0.3%. SSR loci with a length of 18 bp were the most frequent (19.7%, 3,865) followed by those with 15 bp (17.3%, 3388), 12bp (16.8%, 3,293), 16bp (14.6%, 2,860) and 14 bp (12.6%, 2,467). The longest SSR locus was 57 bp (Figure 3C). From 19,596 SSR loci, 205 different repeat motifs, considering sequencing complementary, were identified. The 13 most abundant SSR repeat motifs with different levels of repeats are shown in Table 3. The most abundant motifs were dinucleotide repeat units AG/CT, AT/AT and AC/GT with frequencies of 45.2%, 8.0% and 7.8%, respectively. Among the trinucleotide repeat units, AGG/CCT, AAG/CTT and CCG/CGG were the most abundant with frequencies of 7.1%, 6.1% and 5.3%, respectively (Table 3).

**Table 3:** **Frequency distribution of the 13 most frequent SSR repeat motifs in the *Amorphophallus *transcriptome with different levels of repeats**

**s. no**	**Repeats motif**	**Number of repeats of the motif**										
		**5**	**6**	**7**	**8**	**9**	**10**	**11**	**12**	**13**	**total**	
1	AG/CT	-	2233	1797	1632	1808	1166	217	11	2	8866	45.2%
2	AT/AT	-	518	320	252	206	195	72	10	1	1574	8.0%
3	AC/GT	-	487	325	222	173	200	106	11		1524	7.8%
4	AGG/CCT	800	421	161	10					1	1393	7.1%
5	AAG/CTT	638	358	177	22			1		2	1198	6.1%
6	CCG/CGG	674	261	102	6	2					1045	5.3%
7	AGC/CTG	508	259	128	12		1			1	909	4.6%
8	ACC/GGT	261	128	59	11					1	460	2.3%
9	ATC/ATG	157	86	60	13						316	1.6%
10	ACG/CGT	136	56	24	6					2	223	1.1%
11	AAT/ATT	107	53	24	10			2		3	199	1.0%
12	AAC/GTT	79	29	15	9			1		1	134	0.7%
13	CG/CG	-	55	25	18	3	2	2			105	0.5%

### Development, validation and transferability of SSR markers

Based on combined non-redundant unigene sequences containing 19,596 SSR loci, 10,754 primer pairs (Additional file 1) were designed using Primer3.0 [29]. Primer pairs for the remaining 8,842 loci could not be designed successfully because the sequences flanking the SSR loci were either too short or were not appropriate for designing primers. From the 10,754 primer pairs, 320 were randomly selected for primer synthesis and validation (Additional file 2).

Among the 320 primer pairs, 270 were successful in PCR amplification using genomic DNA from *A. konjac* and *A. bulbifer*. The remaining 50-pair primers failed to generate PCR products, even when the annealing temperature was reduced by 8°C. Of the 270 working primer pairs, 198 yielded PCR products of the expected fragment size. The other 31 primer pairs generated multiple bands that did not include the target bands. In addition, 36 primer pairs amplified fragments larger than the expected size, probably because of the presence of an insertion mutation. Only five primer pair amplified a shorter than expected fragment, suggesting that a deletion mutation had occurred in the amplified region. To test the inter-species transferability, all 230 validated primer pairs, excluding the 31 pairs that generated multiple bands and nine pairs that generated more than 500 bp bands, were screened for polymorphism among 25 individual plants. Thirty five pairs of primers could amplify PCR products from all *Amorphophallus* species (Additional file 3). Twelve pair of primers only produced PCR fragments in *A. bulbifer*; seven pairs (AK-EST-SSR55, AK-EST-SSR63, AK-EST-SSR72, AK-EST-SSR78, AK-EST-SSR83, AK-EST-SSR120 and AK-EST-SSR162) amplified successful from *A. bulbifer* and *A. bulbifer* cultivars; four pairs (AK-EST-SSR21, AK-EST-SSR55, AK-EST-SSR57 and AK-EST-SSR79) amplified successful from *A. bulbifer*, *A. yuloensis* and *A. bulbifer* cultivars; and twelve pairs amplified successful from *A. konjac*. The remaining 161 primer pairs successfully amplified fragments from 3 to 19 species. Therefore, the EST-SSRs developed from *A. konjac* and *A. bulbifer* could be successfully applied to other *Amorphophallus* species, with a transferability rate of 86.9%.

Of 230 primers, 25 primers were monomorphic; the other 205 primers were polymorphic; the proportion of polymorphic primers was 89.1%. From the 205 polymorphic loci, the number of alleles per locus ranged from 2 to 14 alleles. A total of 1,030 alleles were identified, with an average of 5.02 alleles per locus. Across 188 loci, the polymorphic information content (PIC) ranged from 0.10 for AK-EST-SSR207 to 0.90 for AK-EST-SSR4 (Additional file 3), suggesting that the developed EST-SSRs were highly polymorphic. To determine the likely function of these EST-SSRs, they were subjected to BLAST analysis. The results showed that 124 transcriptome sequences from the 205 polymorphic SSR makers shared significant homology to other plants functional loci. The functional annotations of the developed markers are listed in Additional file 3.

### The genetic diversity and cluster analysis in genus *Amorphophallus*

**Figure 4:**
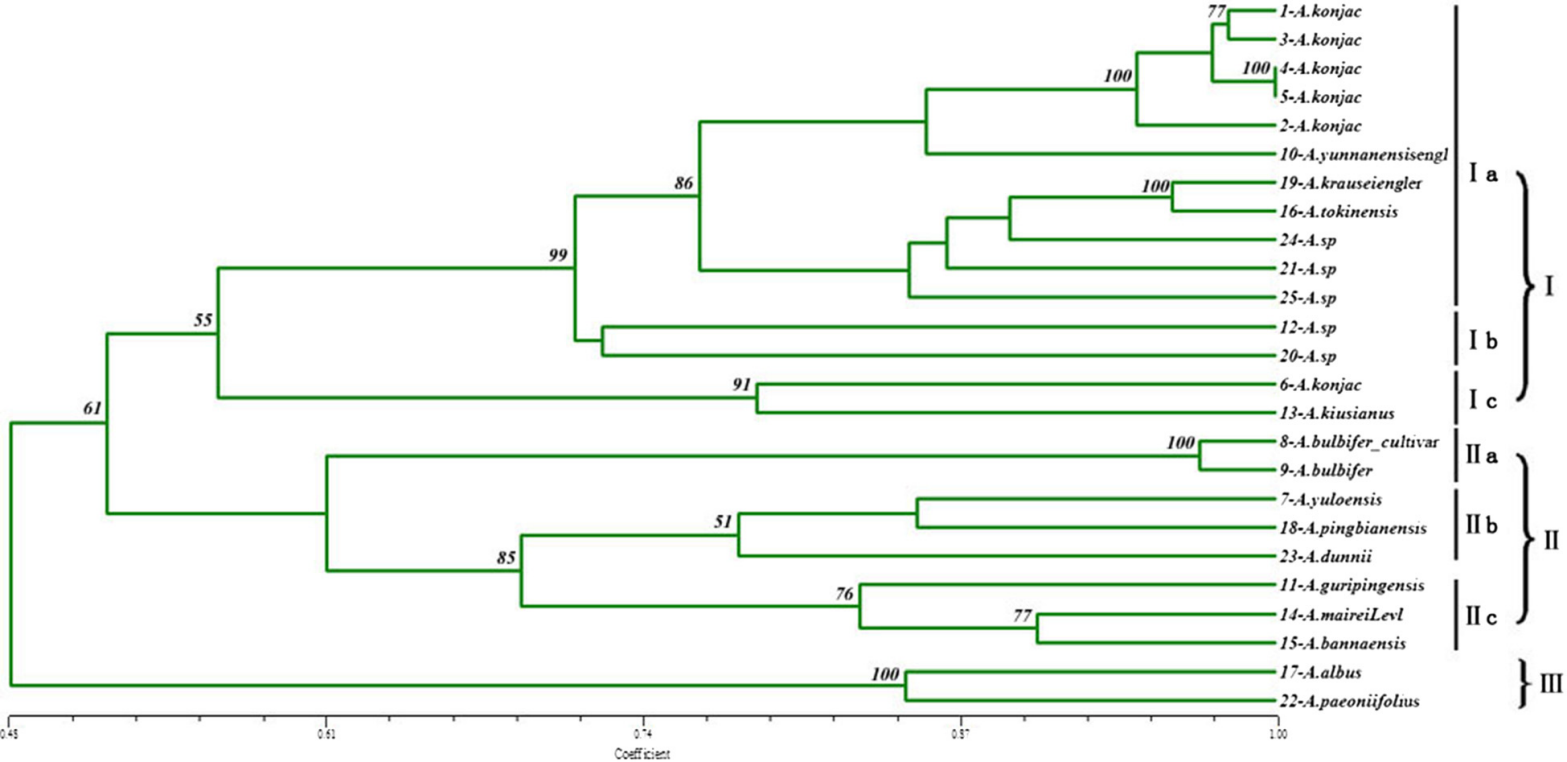
**Genetic relationships among *Amorphophallus *species based on EST-SSR markers.** The dendrogram shows the genetic relationships among 25 individual plants. The scale at the bottom of the dendrogram indicates the level of similarity between the genotypes, bootstrap values (>50) were labeled on the branches from 1000 re-samplings.

The 177 pair primers that yielded clear, highly polymorphic bands were used to assess the genetic diversity in a set of 25 individual plants representing diverse wild unknown species and cultivated genotypes of *Amorphophallus* (Table 4). A phenogram tree based on Jaccard’s similarity coefficients was constructed, which showed three distinct clusters at a cut-off similarity index of 0.55 (Figure 4). Cluster I contained five species and five unknown “species”, and was divided into three sub-clusters: Ia, Ib and Ic, at a cut-off similarity index of 0.71. Sub-cluster Ia comprised two wild *A. konjac*, three *A. konjac* cultivar genotypes, three Yunnan species and three unknown “species”. Sub-cluster Ib comprised two unknown “species” from Chongqing and Yunan, respectively. Sub-cluster Ic comprised one wild *A. konjac* and one *A. kiusianus*. The wild *A. konjac* species from Kunming was distant from the other five *A. konjac* species. Cluster II included seven species and it was divided into three sub-clusters IIa, IIb and IIc, at a cut-off similarity index of 0.69. Sub-cluster IIa comprised one *A. bulbifer* and one *A. bulbifer* cultivar; sub-cluster IIb comprised one Hunan species and two Yunnan species; sub-cluster IIc comprised three species from Yunnan. Cluster III contained *A. albus* and *A. paeoniifolius* from Yunan and Chongqing, respectively.

**Table 4:** **The 25 individual plants (belonging to 14 species and 5 unknown “species**”) **used for validation and the genetic diversity study**

**No**.	**Species**	**Source**	**Characteristics**
1	*A. konjac*	Chengdu, Sichuan	Cultivar
2	*A. konjac*	Fujian	Cultivar
3	*A. konjac*	Enshi, Hubei	Wild
4	*A. konjac*	Enshi, Hubei	Cultivar
5	*A. konjac*	Zhangjiajie, Hunan	Wild
6	*A. konjac*	Kunming, Yunnan	Wild
7	*A. yuloensis*	Kunming, Yunnan	Cultivar
8	*A. bulbifer*	Wuhan, Hubei	Cultivar
9	*A. bulbifer*	Wuhan, Hubei	Wild
10	*A. yunnanensisengl*	Kunming, Yunnan	Wild
11	*A. guripingensis*	Kunming, Yunnan	Wild
12	A. sp*	Kunming, Yunnan	Wild
13	*A. kiusianus*	Kunming, Yunnan	Wild
14	*A. maireiLevl.*	Kunming, Yunnan	Wild
15	*A. bannaensis*	Kunming, Yunnan	Cultivar
16	*A. tokinensis*	Kunming, Yunnan	Wild
17	*A. albus*	Kunming, Yunnan	Cultivar
18	*A. pingbianensis*	Kunming, Yunnan	Wild
19	*A. krauseiengler*	Kunming, Yunnan	Cultivar
20	A. sp*	Chongqing	Wild
21	A. sp*	Chongqing	Wild
22	*A. paeoniifolius*	Chongqing	Wild
23	*A. dunnii*	Changsha, Hunan	Wild
24	A. sp*	Enshi, Hubei	Wild
25	A. sp*	Myanmar	Wild

* *A. sp* means a wild unknown “species” (different variations or genotypes) with no clear classification.

## Discussion

### SSR marker frequency and distribution in *Amorphophallus* transcriptome

Genetic markers are important for studying population genetic structure, diversity analysis and the genetic basis of adaptive traits [30,31]. Deep transcriptome sequencing provides a good resource for the development of SSRs because of the enormous amount of sequence of data that it generates. Markers based on transcriptome sequences are useful for the detection of functional variation and gene associated genetic analysis [32]. Based on the 135,822 non-redundant unigene sequences, a total of 19,596 SSRs were identified. About 11.8% of the transcriptomic sequences possess SSR loci. This rate is higher than wheat (7.41%), barley (2.8%) and *Epimedium* (3.67%), but lower than coffee (18.5%) [33-36]. The EST-SSRs frequency is dependent on the search parameters for exploring SSR markers, e.g. the repeat length threshold and the number of repeat motifs unit. In our study, the mononucleotide repeat motifs were excluded for identifying SSRs because they can result from sequencing errors. In addition, the software used to detect SSR loci can affect the SSR frequency. Some tools can detect imperfect SSRs, e.g. Sputnik, while SSRIT and MISA (used here) can only detect perfect SSRs. The distribution density in *Amorphophallus* is one SSR loci per 3.63 kb, compared with 3.4 kb in rice, 5.4 kb in wheat, 7.4 kb in soybean, 14 kb in Arabidopsis, and 20 kb in cotton [34,37]. The difference in SSR density could partially depend on the minimum length of the SSR repeat motif [24]. In addition, Varshney *et al*. assumed that the high frequency of SSR in rice EST sequences might be due to its small genome size [38].

Dinucleotide repeat motifs are the most frequent repeat type among the *Amorphophallus* unigenes analyzed, representing 61.6% of the SSR loci identified, followed by Trinucleotide (30.3%), Tetranucleotide (5.6%), Pentanucleotide (1.5%) and Hexanucleotide (1%) (Figure 3A). This is consistent with the EST-SSRs distributions reported in pigeonpea, peach, spruce, pumpkin, kiwifruit and coffee, where the dinucleotide motif is the most frequent [39-43]. As shown in Table 3, the most abundant dinucleotide repeat motif was AG/CT (45.2%) in our transcriptome sequences. The same results were also found in other plant species [44-48]. The most abundant trinucleotide repeat motif was AGG/CCT (7.1%), closely followed by AAG/CTT (6.1%). These results agree with the report from Poncet *et al.*[47]. Unlike the results obtained in *Amorphophallus*, the most frequency trinucleotide repeat motifs were AAC/TTG in wheat, AAG/CTT in soybean and *Epimedium sagittatum*, CCG/GGC in maize, barley and sorghum [36,38,49-51]. The previous studies suggested that the trinucleotide AAG/CTT is a common motif and that CCG/CGG is very rare in dicotyledonous plants. Interestingly, the trinucleotide CCG/CGG motif is common in monocots [52]. In this study, the trinucleotide CCG/CGG motif (5.3%) is the third most common repeat type, closely followed by AGG/CCT (7.1%) and AAG/CTT (6.1%). These results strongly support the view that the abundance CCG/CGG motif is a specific feature of monocot genomes, which could have resulted from the high GC content and consequent codon usage bias [53,54].

### Polymorphism and genera transferability of SSR markers

EST-SSRs are very useful and attractive because they are located in coding regions of the genome. Therefore, they often show a high degree of transferability to related species [55-57]. In this study, 320 designed primer pairs were used for validation of the EST-SSR markers in *Amorphophallus*, 270 primer pairs (84.4%) yielded amplicons in *A. konjac* or *A. bulbifer*. This result was similar to the success rate of 60-90% amplification in previous studies [41,44,48,52,55,58,59]. From 230 primer pairs, we obtained 205 polymorphic EST-SSR markers within 25 samples, with the polymorphic proportion of 89.1%. Compared with other plants, the polymorphic ratio of EST-SSRs in *Amorphophallus* is very high [51,60]. Using these polymorphic markers, 1,030 alleles were identified, with an average of 5.02 alleles per locus in 25 individual plants. PIC value ranges from 0.10 to 0.90. Santosa *et al.* reported 19 polymorphic SSR markers with an average of 14.5 alleles per locus in *A. paeoniifolius*[15]. Santosa *et al.* also reported ten SSR markers with an average of 0.403 gene diversity (PIC) in 61 *A. paeoniifolius* individual plants [11]. The transferability of EST-SSRs among different related genera has been reported in many crop plants. Ellis *et al.* summarized the transferablility of EST-SSRs and showed that the ratio of EST-SSRs cross-genera transferability ranged from 10% to 90% [61]. In this study, the *Amorphophallus* species had a high transferability rate (86.9%). This high success rate indicated that the different species in the genus *Amorphophallus* may evolutionarily closely related.

### Evaluation of genetic relationships among different species of *Amorphophallus*

Collection of germplasm has been carried out in China, India and Japan. However, until now, evaluation of the genetic diversity of *Amorphophallus* germplasm has been limited. In this study, three major groups were identified at a cut-off similarity index of 0.55. The level of genetic similarity (0.48-1.00) was revealed in the 25 individual plants, which included 14 species and 5 wild unknown “species”, using 177 polymorphic primers. The genetic variation between *A. konjac* and *A. paeoniifolius* was the highest among species in *Amorphophallus*. The intra-specific variation in *A. konjac* species is higher than that of some inter-species, and the *A. konjac* wild genotypes were clustered with cultivated genotypes. These results implied that *Amorphophallus* has a rich genetic diversity; the variation between wild and cultivar genotypes in *A. konjac* species was very small. This may be because the cultivars have been directly derived from the wild species without systematic genetic breeding. In addition, five unknown *Amorphophallus* “species” were used to evaluate their taxonomic status. 21-*A. sp* from Chongqing, 24-*A. sp* from Hubei and 25-*A. sp* from Myanmar were clustered in one group with *A. krauseiengler* and *A. tokinensis*. 20-*A.sp* and 12-*A.sp* comprised one group: sub-cluster Ib. Evaluation of the genetic status of these unknown species is valuable for utilizing new resources and for breeding. Sedayu *et al.* reported that the molecular phylogeny of the genus *Amorphophallus* contained 69 taxa, based on a combined analysis of trnL, rbcL and LEAFY second intron sequences, which reflected the biogeographical distribution of *Amorphophallus*[13]. Grob *et al.* also reported the phylogeny of 46 *Amorphophallus* using the nuclear FLORICAULA/LEAFY second intron [12]. Our results complement and support these previous reports.

## Conclusions

A large-scale transcriptome dataset with 135,822 unigenes from *A. konjac* and *A. bulbifer* is reported. A total of 10,754 primer pairs were successfully designed based on the transcriptome sequences. Three hundred and twenty primer pairs from the combined non-redundant unigenes of the two sequenced *Amorphophallus* species were validated. Using the validated primers, the diversity of 25 individual plants containing 14 species and 5 wild unknown “species” was analyzed. The developed SSR markers are a valuable resource for studying genetic diversity, linkage mapping, and germplasm characterization analysis in *Amorphophallus* (Araceae). The ESR-SSR markers were developed based on conserved expressed sequences; therefore, they may be applied to other species because of their high transferability. To the best of our knowledge, this is the first attempt to exploit a transcriptome database and develop a large set of SSR markers in *Amorphophallus*.

## Methods

### Plant materials

Tender leaves of two species in the genus *Amorphophallus* (*A. bulbifer* and *A. konjac*) were collected for RNA extraction and transcriptome sequencing. Twenty-five individual plants were used for DNA extraction, PCR amplification, SSR marker validation and diversity analysis. All materials in this study were planted in the Wuhan University nursery (Wuhan, Hubei province, China). Detailed information for the plant materials is listed in Table 4.

### RNA extraction, reverse transcription and sequencing

Total RNA was isolated using TRIzol kit (Invitrogen) according to the manufacturer’s instructions and then purified to exclude the rRNA or tRNA for enrichment of mRNA using magnetic oligo(dT) microspheres. Fragmentation buffer was added to break the mRNA into short fragments (200nt-700nt). The mRNA fragments were reverse transcribed using Power-script™II with random hexamers primers. cDNA were then synthesized using DNA polymerase I. Double-stranded cDNA (ds cDNA) were purified using QiaQuick PCR kit and eluted with EB buffer. After ds cDNA end repair, poly (A) tailing and the addition of sequencing linkers, the correctly sized fragments were purified by Agarose Gel Electrophoresis gel and amplified using sequencing primers. The PCR products were sent to Illumina for HiSeq™ 2000 sequencing.

### Development of SSR markers and primer designing of two *Amorphophallus* species

Two sets of expressed sequences reads were generated by high-throughput transcriptome sequencing from cDNA libraries of the two *Amorphophallus* species. High quantity filtered transcriptome reads were obtained by Illumina HiSeq™ 2000, and contigs were generated for the two species separately by *de novo* assembly using Trinity tools [28]. A non-redundant dataset of unigene sequences was then created using paired-end reads, which ensures the distance between different contigs from the same transcriptome. This unigene dataset was used for detecting SSR loci by a Perl script known as MicroSAtellite (MISA, http://pgrc.ipk-gatersleben.de/misa) and primers were designed using Primer 3.0 [29]. The SSR loci were only considered to contain two to six nucleotides motifs with a minimum of 6,5,4,4 and 4 repeats, respectively. Mononucleotide repeats were excluded.

The parameters for designing PCR primers were as follows: (1) primer length ranging from 18 to 28 bases; (2) PCR product size range of 100 to 280 bp; (3) melting temperature between 57°C and 63°C, with 60°C as the optimum annealing temperature; (4) a GC content of 40%-60%, with an optimum of 50%.

### Plant DNA extraction, PCR conditions and separation of SSR markers

Plant DNA was isolated from leaf samples of 25 individuals (Table 4) according to CTAB method [62]. Electrophoresis through a 1.5% agarose gel was used to check DNA integrity. The SSR markers were initially tested for amplification using DNA from the two species used to generate the transcriptome sequences to optimize the annealing temperature. PCR amplifications were carried out using MyCycler™ Thermal Cycler (Bio-RAD) in a final volume of 15 ul. Each reaction tube contains 1.5 μl of 10 × PCR buffer, 0.6 μl of dNTP (10 mM), 1.0 μl of Mgcl_2_ (20 mM), 1 μl of each primer (10 pmol), and 2 μl of genomic DNA (50 ng), 0.5 μl of Taq DNA polymerase (1 U, Biostar). The PCR reaction program was: DNA denaturation at 94°C for 4 min; 36 cycles of 94°C for 1 min, 60°C for 1 min, 72°C for 1 min.; and 72°C for 7 min as a final extension. The primers that were not successful for amplification or produced multiple bands were reanalyzed using the touchdown PCR method with 1°C increments. The optimized SSR primers were used to amplify DNA from 25 plant individuals for genetic diversity analysis. The PCR products were separated using 6% polyacrylamide gel, 0.5 × TBE buffer in an EC160 DNA Sequencing System (Thermo EC). Gels were stained with silver nitrate, following the protocol described in Han *et al.*[63]. A PBR322 DNA marker ladder (Tiangen Biotech, Beijing co., LTD.) was used for assessing the length of the DNA bands.

### Genetic analysis and data scoring

The PCR products were scored manually in binary format, with the absence of a band being scored as 0 and its presence as 1, thus generating a binary matrix. The polymorphism information content (PIC) of each EST-SSR primer pair was calculated using the formula as following:

$$\mathrm{PIC}=1-\sum_{i=0}^{n}Pi^{2}$$

where, *Pi* is the frequency of the *i*^*th*^ allele for a given SSR loci, and *n* is the total number of alleles detected for the SSR markers [64]. The genetic similarity between any two individuals was accessed based on Jaccard’s similarity coefficient. All 25 individuals were clustered using UPGMA analysis and the SHAN clustering program by NTSYS-pc v2.10 t [65]. Bootstrapping analysis (1000 re-samplings) was carried out using software FREETREE V.0.9.1.50 [66]. Bootstrap values over 50 are considered significant and provided on the dendrogram.

## Competing interests

The authors declare that they have no competing interests.

## Authors’ contributions

PC and YYN collected plant materials and performed the SSRs validation and diversity analysis. DY and HZL designed the study. The transcriptome data processing and writing of this manuscript were completed by ZXF. In addition, YCZ participated in data analysis and material collection. All the authors read and approved the final manuscript.

## Supplementary Material

Additional file 1 **Primer sequences for SSR loci.** SSR primes were designed using Primer 3.0. The sequence ID, Unigene Source, SSR type, SSR motif type, motif size, location of the SSR, location of both primers, primer sequences, length of primers, melting temperature, and amplicon product size are provided for each locus. Click here for file

Additional file 2 **Characterization of 320 primer pairs amplification in 25 individual plants.** Primer name, sequence ID, repeat motif, sequence of both primers, melting temperature and expected size (bp) are indicated. Click here for file

Additional file 3 **Details of 230 validated EST-SSR markers and predicted functions of their genes, based on BLASTX.** Primer name, sequence ID, repeat motif, expected size (bp), objective size (bp), number of species from which it was successfully amplified, N. alleles, PIC value, Swissprot-annotation, and COG-Function-Description are indicated. Click here for file
